# Cost-Effectiveness of Lifestyle Counselling as Primary Prevention of Gestational Diabetes Mellitus: Findings from a Cluster-Randomised Trial

**DOI:** 10.1371/journal.pone.0056392

**Published:** 2013-02-15

**Authors:** Päivi Kolu, Jani Raitanen, Pekka Rissanen, Riitta Luoto

**Affiliations:** 1 UKK Institute for Health Promotion, Tampere, Finland; 2 School of Health Sciences, University of Tampere, Tampere, Finland; 3 National Institute for Health and Welfare, Department of Children, Young People and Families, Helsinki, Finland; Fundación para la Prevención y el Control de las Enfermedades Crónicas No Transmisibles en América Latina (FunPRECAL), Argentina

## Abstract

**Aims:**

The aim was to evaluate the cost-effectiveness of primary prevention of gestational diabetes mellitus (GDM) through intensified counselling on physical activity, diet, and appropriate weight gain among the risk group.

**Materials and Methods:**

The cost-effectiveness analysis was based on data from a cluster-randomised controlled GDM prevention trial carried out in primary health-care maternity clinics in Finland. Women (*n* = 399) with at least one risk factor for GDM were included. The incremental cost-effectiveness ratio (ICER) was calculated in terms of birth weight, 15D, and perceived health as measured with a visual analogue scale (VAS). A bootstrap technique for cluster-randomised samples was used to estimate uncertainty around a cost-effectiveness acceptability curve.

**Results:**

The mean total cost in the intervention group was €7,763 (standard deviation (SD): €4,511) and in the usual-care group was €6,994 (SD: €4,326, *p* = 0.14). The mean intervention cost was €141. The difference for costs in the birth-weight group was €753 (95% CI: −250 to 1,818) and in effects for birth weight was 115 g (95% CI: 15 to 222). The ICER for birth weight was almost €7, with 86.7% of bootstrap pairs located in the north-east quadrant, indicating that the intervention was more effective and more expensive in birth weight terms than the usual care was. The data show an 86.7% probability that each gram of birth weight avoided requires an additional cost of €7.

**Conclusions:**

Intervention was effective for birth weight but was not cost-effective for birth weight, 15D, or VAS when compared to the usual care.

**Trial Registration:**

ISRCTN 33885819.

## Introduction

There is a trend of increasing prevalence of gestational diabetes mellitus (GDM) [1], [2]. In Finland, the prevalence of GDM was 10.3–11.2%, according to the Medical Birth Register, in 2004–2006 [3]. GDM, which is a term for diabetes first appearing during pregnancy and diagnosed with a glucose tolerance test [4], is associated with an increase in total health-care costs [5]. In addition, according to previous studies, women with GDM had 18% higher delivery-stay costs than did women without GDM [6]. Antenatal outpatient costs due to visits for primary health care (antenatal health care) and specialist health care (visits to a regional or university hospital) were 30.4% higher among women with GDM than among those without GDM diagnosis [7]. However, being overweight pre-pregnancy, as a risk factor for GDM, was associated with more inpatient and outpatient visits during pregnancy and the postnatal period [8], [9] than among women of normal weight. To control health-care costs, one should focus on prevention of GDM via lifestyle counselling, because low physical activity, being overweight, and GDM in an earlier pregnancy are correlated with risk of GDM [4], [10]–[12], though data from the Medical Birth Register for 2006 indicate that 2.4% of women with a GDM diagnosis had no GDM risk factors [7].

GDM affects the health of mother and foetus: it increases the mother’s risk of hypertensive disorders during pregnancy, such as pre-eclampsia, and of caesarean delivery [13], [14] but also the risk of type 2 diabetes and metabolic syndrome later in life [4], [14]. In addition, GDM is associated with prenatal and postnatal complications [11], such as shoulder dystocia and risk of macrosomia [11], [13], [14], and high birth weight increases the risk of metabolic syndrome for the newborn and impaired glucose intolerance later in life [14].

Health-promoting interventions with dietary advice, blood-sugar monitoring, and insulin therapy have proved to be effective when compared to routine care to prevent newborns’ perinatal complications; improve the quality of life of women with GDM [15]; and decrease risks of macrosomia, shoulder dystocia, and caesarean delivery in women with mild GDM [16]. In addition, prevention programmes with insulin therapy, if needed, decreases both the health-care costs related to the stay in hospital for women with diabetes during pregnancy [17] and serious perinatal complications [18]. There have been only a few cost-effectiveness studies related to GDM, one on treatment of mild GDM [19] and the other evaluating the cost-effectiveness of an exercise programme for a GDM-risk group, measured in terms of blood glucose levels, insulin-sensitivity, birth weight, and pregnant women’s quality of life [20]. As no evidence of cost-effectiveness of lifestyle counselling was found in that study, by Oostdam et al. [20], we hypothesised that intervention is not cost-effective. We have reported effectiveness results of our earlier GDM trial showing favourable changes in diet composition and proportion of large-for-gestational-age newborns [21], [22]. The aim of the present study was to assess the cost-effectiveness of primary prevention of GDM through intensive dietary and physical-activity counselling among women with a risk of GDM.

## Methods

### Study Design and Intervention

The cost-effectiveness analysis was based on a cluster-randomised GDM prevention trial (*n* = 399) conducted at maternity clinics in Finland from 2007 to 2009 (trial registration: ISRCTN33885819) [21], [23]. The economic evaluation data’s collection was implemented during the original RCT study, whose aim was to assess the effectiveness of primary prevention of GDM via intensive dietary and physical-activity counselling among women with a risk of GDM [21], [23]. On the basis of voluntary involvement, 14 maternity clinics were grouped into matched pairs on the basis of number of births, socio-economic status, incidence of GDM, and size of the area’s population. Each set of seven clusters was randomised to an intervention clinic or usual-care clinic, in a process done by computer at the UKK Institute for Health Promotion Research. The reason for randomisation of municipalities instead of nurses or pregnant women was to avoid contamination.

On pregnant women’s first visits to the antenatal clinic, public-health nurses recruited all women who met the criteria and were willing to participate, up to 12 weeks’ gestation. An inclusion criterion was to have at least one of the following GDM risk factors: BMI ≥25 kg/m^2^, GDM or any sign of glucose intolerance, a macrosomic newborn (≥4,500 g) in any earlier pregnancy, type 1 or 2 diabetes in first- or second-degree relatives, and age ≥40 years. The exclusion criteria were a pathological value in a baseline oral glucose tolerance test (OGTT) at 8–12 weeks’ gestation (blood glucose >5.3 mmol/l fasting, >10.0 mmol/l one-hour, or >8.6 mmol/l two-hour), type 1 or 2 diabetes before pregnancy, inadequate proficiency in the Finnish language, age <18 years, twin pregnancy, and physical limitations preventing physical activity.

### The Intervention Group

The intervention programme was developed by the NELLI research group, which consisted of physicians and experts in physical activity and nutrition, as described in the national nutrition and physical-activity recommendations. In addition, the feasibility of the intervention programme was tested in a pilot study [24], [25]. The trial involved five booster visits out of the 11–15 recommend antenatal-care visits from week 8–12 of gestation to 37 weeks’ gestation [26].

The public-health nurses focused on individualised dietary and physical-activity counselling based on national physical-activity and diet recommendations, personalised goals, and regular follow-up of targets but also recommendations for appropriate gestational weight gain [23]. Women who were at least a little physically active and had an uncomplicated pregnancy and a history of at least some physical activity were encouraged to undertake at least 150 minutes of moderate or vigorous leisure-time activity three days a week [27]–[29]. Participants tracked the amount of weekly physical activity in a physical-activity diary, which was checked regularly by a physical-health nurse. Women were briefed to report the type of sport/activity and the intensity and duration of the physical-activity session. In addition, the public-health nurse asked about adverse events related to physical activity, such as vaginal bleeding, major contractions, dizziness, headache, chest pain, and muscle weakness, every fifth visit [23], [30].

Moreover, women in the intervention group were offered the opportunity to participate in five sessions including theory and practice of various forms of physical activity, under the instruction of a physiotherapist. Because of the geographical spread of the seven antenatal clinics that had an intervention group, a two-hour themed group session was held at each antenatal clinic [23].

Alongside physical activity, the dietary counselling with recommendations related to gestational weight gain was the main theme of the counselling, in which the aim was to help the participants achieve a diet containing <10% saturated fats, 5–10% polyunsaturated fats, total fat (saturated, monounsaturated, polyunsaturated, and trans fatty acids) accounting for 25–30% of one’s total energy intake, and 25 to 35 grams of fibre per day. A structured notebook for public-health nurses covering themes of nutrition and physical activity ensured that the intervention was administered in a systematic way. In addition, the public-health nurses had a detailed written plan for the content of each counselling session, in which the nurse remarked on the mode of realisation and time of counselling. The aim was to carry out counselling based on the participant’s current aims and her opportunities for and barriers to making the changes.

Information on medication and the number of visits for primary and specialist health care was obtained from maternity cards completed by the public-health nurse at the maternity clinic. Information on visits to a diabetes nurse or a dietician was collected from questionnaires completed by the mothers at the beginning of the pregnancy (at 8–13 weeks’ gestation) and at the end of pregnancy, at 36–37 weeks. The information on the number of the mother’s inpatient days at the hospital before and after the standard delivery stay, the mode of delivery, the ICD-10 diagnosis code of the mother and newborn, and the number of days in hospital for the newborn were obtained from the Medical Birth Register and the Registers for Social Welfare and Health Care HILMO. The former includes, in addition to pregnancy and delivery, information on neonates until they leave the hospital, up to the seventh day after birth. There were 21 dropouts from the intervention group (11.0%).

### The Usual-care Group

The women at the control maternity clinics received routine care, and no extra counselling beyond the usual care or group exercises was arranged. However, routine maternity care does include some dietary and physical-activity counselling in accordance with national guidelines, as shown in our pilot study [27], [28]. The dropout rate in the usual-care group was 8.2% (16 participants withdrew).

### Ethics Statement

This study was approved by the medical ethics committees of the Pirkanmaa hospital district (R06230). All participants in the original GDM prevention trial who gave verbal consent to being included in the study on the first contact with the maternity clinic signed the written informed consent form at the first maternity visit. All eligible women gave informed consent for later registry linkage, for provision of the missing cost data. In addition, at that time, the participant’s right to refuse to be involved in the study without giving reasons and the fact that all information would be treated in confidence were emphasised. For newborn-care cost data, additional written informed consent was not needed, because the information is included in maternal data until the age of seven days.

### Economic Evaluation

The economic evaluation included health-care costs for the municipality, costs borne by the patient, and productivity costs from the societal perspective. Because the travel expenses and time costs related to the use of health services were assumed to be minor, they were not included in the calculation. Costs were calculated from the beginning of pregnancy until the last day the mother and newborn spent in hospital after the birth, which was a precise cut-off point for economic evaluation.

Costs of days in hospital preceding and following delivery were determined from the number of inpatient days, including the standard inpatient charge of 30 euros a day but not including delivery costs. Delivery costs were calculated separately and depended on the mode of delivery: vaginal delivery, instrumental delivery, elective caesarean section, or emergency caesarean section. The delivery-related unit cost includes the mean number of inpatient days, delivery-operation costs in line with the mode of delivery, the salary costs of obstetrics staff (including administrative expenses), medication, and the cost of neonatal care in cases with no ICD diagnosis. Thus, in the case of rooming-in, the costs of the newborn baby’s care were included in the mother’s delivery unit cost. The newborns’ hospital stays were calculated separately according to ICD-10 diagnosis code in cases wherein the newborn needed immediate neonatal services because of a disease involving organic complications. The cost evaluation for women’s inpatient days preceding and following delivery included only those hospital days which related to pregnancy or GDM immediately after delivery.

Information about medication and the number of visits to primary and secondary care was obtained from the maternity cards completed by the public-health nurse at the maternity clinic. Information on visits to a diabetes nurse or a dietician was collected from questionnaires completed by mothers at the beginning of the pregnancy (weeks 8–13 of gestation) and at the end of pregnancy (weeks 36–37). The data on the number of the mother’s inpatient days before and after the standard delivery stay, the mode of delivery, the ICD-10 diagnosis code of the mother and newborn, and the number of hospital days for the newborn were obtained from the Medical Birth Register and the Registers for Social Welfare and Health Care HILMO.

Primary health-care costs were based on the average national unit costs for health care [31]. The costs of visits for specialist health care, visits to a diabetes nurse or a dietician, mode of delivery, inpatient days, and neonatal services in specialist health care were estimated from the costs at the Tampere University Hospital, which was the delivery hospital in 91% of cases. The reason for using costs of a specific hospital instead of average health-care costs was to gain the benefit of more specific unit-cost information [31], [32]. However, the unit costs of the university hospital were consistent with average national unit costs: the mean costs of many units in primary health care and hospitals in specialist health care at both regional and university hospitals [31]. Unit costs were entered at the price level for 2009, in euros. The unit costs of outpatient and inpatient obstetric care included salary costs and the administrative and laboratory expenses.

Insulin unit costs were calculated for a period of 2.5 months and included health-insurance reimbursement. Insulin costs were calculated for a period of 2.5 months and included the insurance reimbursement. According to the Finnish national guidelines, insulin treatment should be started, if needed, in the 30th week of gestation and continue until delivery [33]. Information on oral glucose tolerance test (OGTT) as a diagnostic test for GDM, was based on Medical Birth Register and were taken at 8–12 and 26–28 weeks’ gestation [23]. Intervention costs included the cost of the supplemental public-health nurse’s work contribution, which consists of time for planning the implementation, nutrition, and physical activity counselling; contacts by telephone; and data-collection time. Supplemental physiotherapist’s work consisted of implementation of physical-activity group meetings, including salary expenses.

Productivity loss was evaluated by means of self-reported information on absence from part- or full-time work, collected via a questionnaires every trimester. Salary costs were based on women’s average national monthly salary scales in 2009 [34], multiplied by 1.3 to include related expenses. The cost calculation assumed that there were 220 workdays a year.

### Outcome Measurements

Information on weekly physical activity, quality of life (15D), and VAS data were collected via questionnaires completed by mothers at the beginning of the pregnancy (at 8–13 weeks’ gestation) and at the end of the pregnancy (at 36–37 weeks). Amount of weekly leisure-time physical activity was given in terms of a typical week during the past month, with the physical activity grouped into one of three separate intensity categories (hard, moderate, and light) [23].

The quality-of-life evaluation was based on the standardised 15D questionnaire, which is a validated instrument for measuring health-related quality of life (HRQoL) with five alternatives for each dimension [35], [36]. The 15D involves 15 separate dimensions: mobility, vision, hearing, breathing, sleeping, eating, speech, excretion, usual activities, mental function, discomfort and symptoms, depression, distress, vitality, and sexual activity. In generation of the overall HRQoL score, the 15 dimensions are covered by a single index number, from 0 to 1 [36]. In addition, subjective effects of intervention were measured by horizontal VAS, a numeric rating scale from 0 to 10 cm to measure perceived health. The effectiveness of the lifestyle intervention was judged in terms of children’s birth weight, because macrosomia may be connected with increased costs, due to a higher number of complications [37]. Moreover, study of birth weight was a secondary outcome.

Earlier studies found a connection between GDM and perceived poor general health during pregnancy [38], while Halkoaho et al. [39] found no difference in quality of life as measured by 15D after delivery explained by GDM. Information on quality of life and perceived health was obtained from questionnaires at the first maternity-clinic visit (8–13 weeks) and at the end of pregnancy (36–37 weeks). Information on birth weight came from the maternity card or, if the maternity card was missing, the Finnish Medical Birth Register. Also, GDM prevention study data were combined with data from Registers for Social Welfare and Health Care HILMO via the identity code of each participant.

### Statistical Analysis

Descriptive information is given in terms of arithmetic means and standard deviations or as frequencies and percentages. Differences in costs between the groups are reported as means and SDs. The costs were non-normally distributed, so differences between groups were analysed via a non-parametric bootstrap approach. The conventional bootstrap approach is flexible but assumes that the data are independently and identically distributed. The assumption of independence does not hold in cluster-randomised trials (CRTs); therefore, the conventional bootstrap method has to be extended to recognise the clustering inherent in a CRT. To account for clustering and correlation between costs, we used the non-parametric two-stage bootstrap (TSB) method with shrinkage correction [40]. This procedure requires shrunken cluster means and standardised individual-level residuals to be calculated before any resampling. Bootstrap datasets are then constructed through combination of resampled shrunken means with resampled individual-level residuals.

We used a modified version of Davison and Hinkley’s original resampling procedure, proposed by Gomes et al. [41]. In the modified algorithm, shrunken cluster means and standardised residuals are calculated as before, but each cluster mean is now combined with individual residual drawn from the same cluster; i.e., this modified algorithm also allow for unbalanced clusters. The following steps describe the non-parametric TSB algorithm used, for 5,000 resamples:

1. For *i* in 1 to *n_j_* (mothers in cluster *j*)
2. For *j* in 1 to *M_k_* (clusters in group *k*)
3. For *k* in 1 to 2 (group)
4. Calculate shrunken cluster means,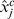 and 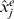, for cost and effect: 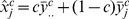, where *c* is given by 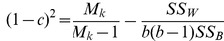 and *SS_W_* = within-sum of squares, *SS_B_* = between-sums of squares, and *b* = average cluster size (harmonic mean)
5. Calculate standardised individual-level residuals, 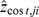 and 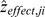, for cost and effect: 
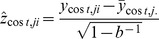
, where 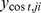 is the observed cost for the *i*th individual in cluster *j* (similar calculations are applied for effects and separately for the intervention and usual-care group)
6. Randomly sample (with replacement) *M_k_* pairs of cluster means, 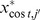 and 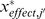, from the shrunken cluster means calculated in step 4
7. Within each resampled cluster, randomly sample (with replacement) 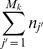 pairs of standardised residuals (step 5), 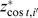 and 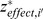, where 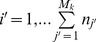

8. Reconstruct the sample (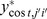 and 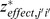) by adding the shrunken cluster means from step 6 and the standardised residuals from step 7; that is, 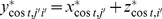, where 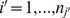 and likewise for effects (call it a ‘synthetic’ sample)
9. Repeat steps 4 to 8 for the intervention and usual-care group, then stack these ‘synthetic’ samples into a single bootstrap sample
10. Replicate steps 6 to 9 *R* times to construct *R* bootstrap samples

Cost-effectiveness was expressed in terms of the incremental cost-effectiveness ratio, which indicates the amount of money required to decrease children’s extra weight or increase the mother’s perceived health as measured by VAS. The uncertainty around the point-estimate ICER was also evaluated via a non-parametric TSB technique with 5,000 replications, as described above. The 95% confidence intervals for mean cost and effect differences between the intervention and usual-care group were calculated via a bias-corrected and accelerated (BCa) method [42]. The coverage error for intervals of a conventional percentile approach is substantial if the distribution of mean cost or effect difference is not nearly symmetric around the observed value. The BCa method is a more reliable percentile approach. The BCa interval is given in percentiles of the bootstrap distribution of differences in means, but the percentiles used are chosen after correction for skewness or ‘acceleration’ â and bias [43].

Multilevel mixed-effects linear regression models (the ‘xtmixed’ command in STATA) were used to test group differences for effectiveness. The goodness-of-fit of the models was evaluated by means of normal probability and residual plots and also tested by the normality of the residuals (Kolmogorov–Smirnov test).

At baseline questionnaire (8–13 weeks’ gestation), six participants (1.5%) had one to six missing 15D dimensions. At the end of pregnancy (i.e., at 36–37 weeks), there were three participants (0.8%) for whom three or six 15D dimensions were missing and all 15D dimensions were missing for 47 participants (11.8%). No-one had missing values in both questionnaires. If six or fewer 15D dimensions were missing (nine participants, 2.3%), imputations for missing values were performed via linear-regression-model technique [36]. First we ran the evaluation algorithm, which generates the new variables *move1*, *see1*, …, *sex1* (the original level numbers were replaced with level values). If, for example, the missing data were related to the *move* dimension, we predicted the levels for that dimension through linear regression analysis. We chose *move1* as the dependent variable and the other level-value variables (*see1*, …, *sex1*) and characteristics (age, education, BMI, and marital status) as independent variables. Unstandardised predicted values from the model were saved, and the missing value of *move1* was replaced with that.

The results were considered to be statistically significant if *p*<0.05. Analyses were performed with SPSS software (version 19.0) and STATA (version 12).

### Sensitivity Analysis

To evaluate the robustness of the findings, we performed sensitivity analysis, using doubled intervention costs.

### Subgroup Analysis

Cost-effectiveness analysis was conducted also for the subgroup of adherent women (*n* = 55), to describe successful lifestyle counselling rather than attendance of counselling etc. Inclusion criteria for this subgroup were weight gain during pregnancy remaining within recommended limits, physical activity exceeding 800 MET minutes at week 36–37 of pregnancy [28], and/or achievement of three of five dietary aims (consisting of intake of dietary fibre, saccharose, total fat, and saturated and polyunsaturated fatty acids [21]).

## Results

### Subjects

Of, in all, 399 pregnant women, 219 (54.9%) were in the intervention group and 180 (45.1%) were in the usual-care group. There were no differences in baseline characteristics or lifestyle habits between these two groups (see Table 1). Neither were there significant baseline differences between the subgroup and the usual-care group.

**Table 1:** Characteristics (mean ± SD or frequency and percentage).

	Intervention group	Usual-care group	Subgroup*	*p* value†
	*n* = 219	*n* = 180	*n* = 55	
Age	29.5±4.8	30.0±4.7	29.2±4.6	0.27
BMI	26.2±4.9	26.4±4.4	26.7±5.9	0.69
Primiparousness	103 (47.0%)	73 (40.6%)	24 (43.6%)	0.68
Education level				0.55
Low	73 (33.8%)	60 (34.1%)	18 (32.7%)	
Medium	85 (39.4%)	80 (45.5%)	22 (40.0%)	
High	58 (26.9%)	36 (20.5%)	15 (27.3%)	
Smoking during pregnancy	46 (21.1%)	45 (25.4%)	10 (18.2%)	0.27
Frequency of separate GDM risk factors	1.33±0.53	1.38±0.61	1.36±0.49	0.76

* Inclusion criteria: physical activity >800 MET minutes per week and/or women having fulfilled at least three of the five dietary aims and weight gain during pregnancy remaining within recommended limits.

† The *p* value was tested between the subgroup and usual-care group.

### Costs

The mean total cost in the intervention group was 11.0% higher than in the usual-care group (*p* = 014) (see Table 2). Mean cost per person in the health-care interventions was €141, which was only 1.8% of the total costs for the intervention group. Over five intensive lifestyle-counselling sessions, public-health nurses’ work contribution per person was, in total, 2.1 hours more than in usual-care sessions, or 18–36 minutes of additional time per visit for the intervention group. Mean direct costs were only slightly (9.5%) higher in the intervention group than in the usual-care group (€5,769 vs. €5,269, *p* = 0.18) (not shown in table).

**Table 2:** Use of health-care services (mean and SD or frequency and percentage) and costs.

		Intervention group		Usual-care group		
		*n* = 219		*n* = 180		
Direct costs	Unit cost, EUR*	Number of units	Mean costs (EUR)	Number of units	Mean costs (EUR)	^∧^P_TSB_
*Women*						
Number of visits for primary health care	€72/visit	14.5	1,044±220	15.1	1,087±196	0.11
Number of visits for specialist health care	€208/visit†	1.57	326±332	1.79	373±387	0.41
Number of visits to a diabetes nurse	€91/hour†	0.12	10±42	0.04	4±20	0.23
Number of visits to a dietician	€164/hour†	0.02	3±27	–	–	0.25
Laboratory tests (OGTT)	€25/test	2	50	2	50	
Use of insulin/other diabetes medication	€85/2.5 months	7 (3.2%)	3±15	8 (4.5%)	4±18	0.62
Hospital days before and after delivery	€330/day‡	1.37	453±889	1.07	352±620	0.32
Delivery cost to the patient	€30/day	3.38	101±31	3.28	98±36	0.61
Delivery cost to the municipality			2,098±635		2,076±622	0.77
*Newborn*						
Neonatal care cost to the patient**	€30/day	3.56	107±38	3.47	104±43	0.69
Neonatal care cost to municipality			1,574±2,044		1,121±1652	0.081
**Productivity costs**						
Absence from work (€,3470/month)	€189/day	9.8	1,853±3,466	9.1	1,725±3502	0.80
**Costs of health-care intervention**						
Supplemental public-health nurse’s workcontribution per person	€56/hour	2.1 hours	118±0		–	
Supplemental physiotherapist’s work contribution per person	€72/hour		23±0		–	
**Total costs**			7,763±4,511		6,994±4,326	0.14

* Costs are rounded to the nearest euro.

† Including outpatient-care charge (€27 per visit).

‡ Including standard in-patient daily charge (€30 per day) and all costs for municipalities and patients.

** In-patient daily charge (€30 per day) over 1–7 hospital days.

### Effects

The mean birth weight was significantly lower in the intervention group than in the usual-care group (*p* = 0.025) (see Table 3). Meanwhile, in terms of changes in the mother’s perceived health or quality of life between the beginning and the end of pregnancy, the intervention was not statistical significantly effective in comparison to the usual care.

**Table 3:** Effectiveness (means and standard deviation) measured in terms of children’s birth weight, 15D, and self-evaluated health (VAS) among the intervention group, the usual-care group, and adherent women.

	Intervention group	Usual-care group		Subgroup*
	*n* = 216	*n* = 179	*p* value†	*n* = 55
**Birth weight**	3,521±545	3,636±500	0.025	3,509±379
**15D**				
Change between pregnancy weeks 8–13 and 36–37	−0.045±0.06	−0.052±0.06	0.24	−0.04±0.05
**Perceived health** (VAS scale of 0–10 cm)				
Change between pregnancy weeks 8–13 and 36–37	−0.32±1.21	−0.56±1.13	0.061	−0.23±0.89

* Inclusion criteria: physical activity >800 MET minutes per week and/or women having fulfilled at least three of the five dietary aims and weight gain during pregnancy remaining within recommended limits.

† Tested with the intervention and usual-care group.

Note: Missing (intervention/usual-care group): birth weight 0/1, 15D 28/22, VAS 27/23.

### Cost-effectiveness

**Figure 1:**
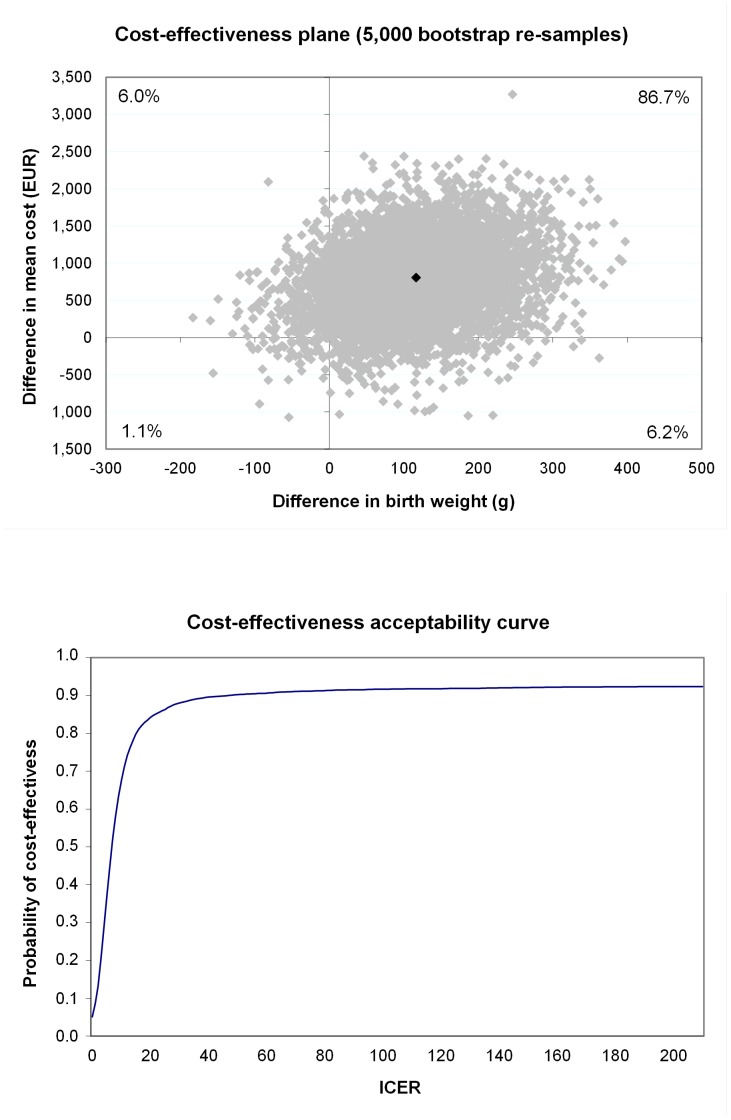
Cost-effectiveness plane (upper) and acceptability curve (lower) for birth weight.

**Figure 2:**
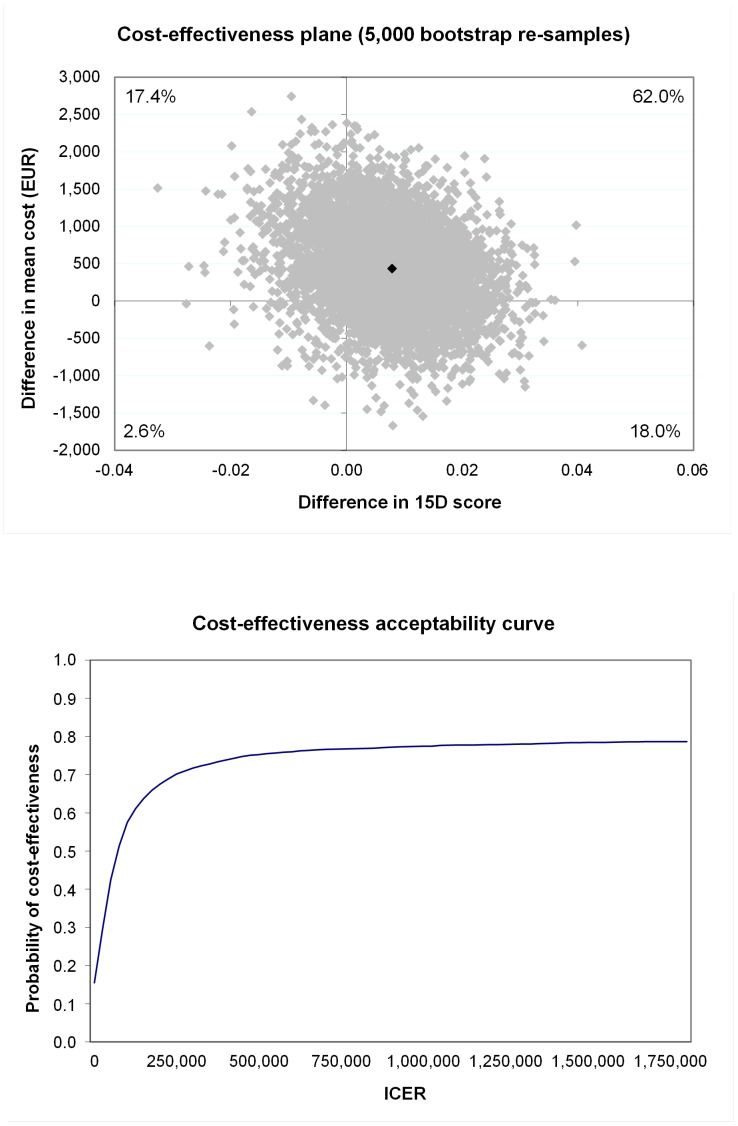
Cost-effectiveness plane (upper) and acceptability curve (lower) for 15D.

**Figure 3:**
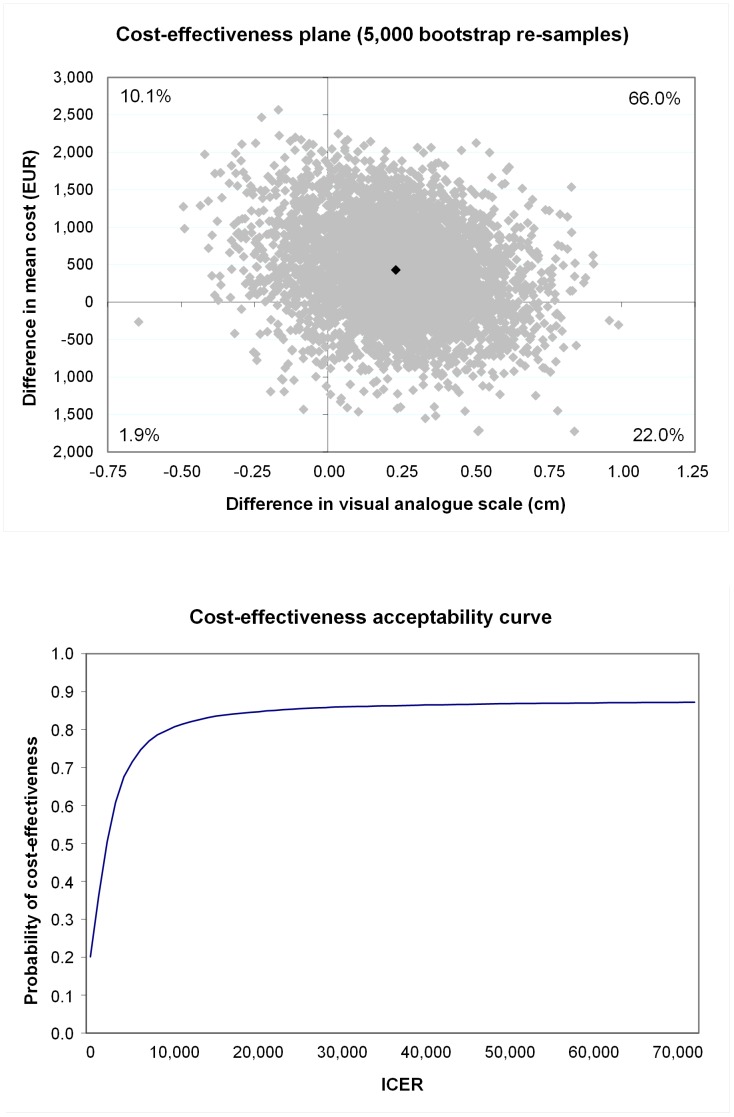
Cost-effectiveness plane (upper) and acceptability curve (lower) for perceived health (VAS).

The incremental cost-effectiveness ratio for birth weight was almost €7 (see Table 4), with 86.7% of bootstrap pairs being in the north-east quadrant indicating that the intervention was more effective and more expensive for birth weight than the usual care (see Table 4). That is, the study indicates 86.7% probability from our data that each gram of birth weight avoided requires an additional cost of €7. The difference in effect measured in VAS terms was 0.24 cm (95% CI: −0.03 to 0.49) between intervention and the usual-care group (see Table 4). The incremental cost-effectiveness ratio for VAS was €1,697, which in this study population represented almost €1,700 in additional costs for achievement of a one-centimetre increase in VAS describing perceived health in pregnant women. The study indicated that intensive lifestyle counselling among GDM-risk groups was not significantly cost-effective as compared to the usual care for birth weight (see Figure 1), quality of life in a 15-dimension questionnaire (see Figure 2), or VAS(see Figure 3), with the ICER for the acceptability curve not reaching a confidence level of 95% for any outcome measures.

**Table 4:** Mean cost and effect differences (95% CI) between the intervention and usual-care group, including incremental cost-effectiveness ratios and cost-effectiveness plane distributions.

	Sample size		Costs (EUR)	Effects	ICER	Distribution of CE plane (%)*			
	Intervention group	Usual-caregroup	Δ of intervention - usualcare (95% CI)	Δ of intervention - usual care (95% CI)		NE	SE	SW	NW
**Total group**									
Birth weight	219	179	753 (−250 to 1,818)	115 (15 to 222)	6.54	86.7	6.2	1.1	6.0
15D	191	158	475 (−723 to 1,609)	0.008 (−0.05 to 0.020)	62,285	62.0	17.4	2.6	18.0
Visual analogue scale	192	157	414 (**−**764 to 1,617)	0.24 (**−**0.03 to 0.49)	1,697	66.0	22.0	1.9	10.1
**Doubled intervention cost**									
Birth weight	219	179	894 (**−**109 to 1,959)	115 (15 to 222)	7.77	89.5	3.4	0.6	6.5
15D	191	158	616 (**−**582 to 1,750)	0.008 (−0.005 to 0.020)	80,760	67.3	12.7	1.6	18.4
Visual analogue scale	192	157	555 (−623 to 1,758)	0.24 (−0.03 to 0.49)	2,274	71.9	16.1	1.4	10.6
**Subgroup** †									
Birth weight	55	179	−514 (−2075 to 1,254)	127 (−16 to 270)	−4.06	22.7	70.7	4.8	2.0
15D	55	158	−772 (−2393 to 978)	0.013 (−0.005 to 0.030)	−60,742	13.0	73.6	10.1	3.3
Visual analogue scale	55	157	−816 (−2421 to 1,019)	0.33 (−0.01 to 0.67)	−2,450	14.0	77.9	6.9	1.2

* 95% CI: 95% confidence interval (‘bias-corrected and accelerated’ method), NE: north-east, SE: south-east, SW: south-west, NW: north-west.

† Inclusion criteria: physical activity >800 MET minutes per week and/or women having fulfilled at least three of the five dietary aims and weight gain during pregnancy having remained within recommended limits.

### Sensitivity Analysis

The sensitivity analysis applied the assumption that the intervention costs would be two times higher (€282). The results were quite similar to those in the basic analysis (see Table 4).

### Subgroup Analysis

The mean total cost per adherent woman in the intervention group was €6,314, which was 19% lower than the mean total cost in the intervention group (not shown in table). Table 3 depicts the change during pregnancy as measured by birth weight, quality of life according to the 15D instrument, and perceived health in the subgroup as compared to the usual-care group. The mean difference between effects in the subgroup and those in the usual-care group for birth weight was 127 g (95% CI: −16 to 270) (see Table 4). The ICER among women who fulfilled the recommendations for good nutrition, physical activity, and appropriate weight gain during pregnancy was €4 for birth weight. In the adherent group, perceived health measured in VAS terms showed a statistically significant effect, unlike birth weight and 15D.

## Discussion

Intensive lifestyle counselling among women at risk for GDM was not cost-effective for birth weight, 15D, or VAS when compared to current practice in antenatal care. Nevertheless, the intervention was slightly more effective than the usual care for effects on birth weight. In addition, because of the children’s lower birth weight, intervention may help to prevent further metabolic syndrome or impaired glucose-tolerance and thus save on further costs of medication and outpatient visits. According to the findings for the subgroup, the intervention was slightly more cost-effective and was correlated with lower health-care costs and children’s lower birth weight in comparison to the usual-care group.

The results of our study were similar to those of Oostdam et al. [20], who found an exercise programme during pregnancy not cost-effective for prevention of gestational diabetes, measured in terms of maternal fasting blood glucose/insulin-sensitivity; neither was the exercise programme cost-effective for changes in quality of life or birth weight. The cost-consequence analysis by Moss et al. [18] in which women with mild GDM were found to use more outpatient services, such as visits to a dietician or diabetes nurse, showed favourable results, unlike our research. The aim of our study was to prevent GDM, while that of Moss and colleagues was to evaluate the effectiveness of mild-GDM treatment. However, the health-care costs of adherent women in the intervention group were significantly lower, and intervention was cost-effective for birth weight, 15D, and perceived health in comparison to the usual-care group. Subgroup analysis among adherent women confirms the importance of individualised and versatile lifestyle counselling at the antenatal clinic that is begun early and involves regular physical activity, moderate weight gain, and recommendation-based nutrition.

The reason for the subgroup analysis was to evaluate the optimal, though unrealistic, situation for the health-promotion intervention: all participants complying with the recommendations. Assessments for the subgroup were performed in the 36th–37th week of pregnancy, which can be an effect of positive changes in lifestyle during pregnancy due to intervention or ability to maintain a healthy lifestyle through maternity. However, evaluation of adherence only at the end of pregnancy may have caused bias. Regular physical activity and weight gain within recommended limits may have long-lasting effects on mothers’ and babies’ insulin-sensitivity, cardiovascular, and metabolic health [29]; therefore, a weakness of our study was that there was information on a participant’s physical activity only at the beginning and at the end of pregnancy. In cases of decreased physical activity in the final weeks of pregnancy, we missed the information on the level and intensity of physical activity. From society’s perspective, a favourable effect on children’s birth weight among women whose case involves risk of macrosomia may bring considerable cost savings later in life, due to avoidance of obesity and of disturbances in glucose metabolism in childhood [44]. Because these consequences cannot be evaluated yet, total cost-effectiveness may be underestimated if one examines pregnancy data only.

The unequal numbers of participants in the clusters may have created inherent bias as a consequence of unequal intake due to public-health nurses’ level of willingness to recruit participants in view of the increased work load, number of pathological OGTT results, or frequency of the GDM risk factors that were inclusion criteria. However, non-parametric two-stage bootstrapping was used to reduce the inherent bias associated with the different number of participants in the intervention and usual-care groups. In addition, the quantity of missing data at the end of pregnancy may also have caused inherent bias, because the reduced sample size may leave the power of the study insufficient for gaining statistically significant findings as to quality of life and perceived health (VAS).

The indicators used to evaluate effectiveness, such as the 15D, may not be effective enough for pinpointing differences between groups. However, according to Moss et al. [18], treatment of mild-GDM women increased health-related quality of life during pregnancy in comparison to the usual care. Pregnancy brings with it many changes in health that may decrease quality of life and perceived health and could reduce capacity to be physically active, yet no pregnancy-specific indicators for quality of life are available. On the other hand, changes are highly individual, and mothers in their maternity period are, in general, very receptive to health education.

A key advantage of the study was that it was embedded in current maternity care, without involving extra visits or arrangements such as research nurses; however, the intervention involved more than two hours in additional counselling sessions over five visits per participant, so it appears that there is some level of additional burden placed on nurses or other providers tasked with administering the intervention components. Intervention costs may have been lower via another mechanism, and implementation in other health-care settings more feasible. The study confirms advantages of lifestyle counselling among the GDM-risk group and may help decision-makers utilise the results of the study in practice.

A weakness of the study was that its findings cannot be generalised to all pregnant women, since we took a risk-group approach. However, the risk factors for GDM, especially being overweight, are quite commonplace in the general pregnant population [45], [46]. There is need for more trials to evaluate cost-effectiveness and find ways to bring about cost-effectiveness of lifestyle counselling in maternity care.
